# Comparison of Alvarado score, appendicitis inflammatory response score (AIR) and Raja Isteri Pengiran Anak Saleha appendicitis (RIPASA) score in predicting acute appendicitis

**DOI:** 10.1016/j.heliyon.2023.e13013

**Published:** 2023-01-16

**Authors:** Muhammad Zeb, Sabir Khan Khattak, Maryam Samad, Syed Shayan Shah, Syed Qasim Ali Shah, Abdul Haseeb

**Affiliations:** a Surgical Resident, General Surgery Ward, Hayatabad Medical Complex, Peshawar, KPK, Pakistan; b Surgical Resident, Orthopedic Ward, Hayatabad Medical Complex, Peshawar, KPK, Pakistan

**Keywords:** Acute appendicitis, Alvarado score, RIPASA, AIRS

## Abstract

**Introduction:**

Acute Appendicitis is the most common surgical emergency encountered in emergency departments. To prevent the rate of negative appendectomies, different systems i.e. Alvarado score and Appendicitis Inflammatory Response Score (AIR) scores were used, but their diagnostic accuracy in Asian population is questionable. Raja Isteri Pengiran Anak Saleha (RIPASA) score has showed promising results in the recent literature. The purpose of this study is to compare the efficacy of Alvarado, AIR and RIPASA scores in the diagnosis of acute appendicitis.

**Methods:**

Alvarado, AIR and RIPASA scores were prospectively applied to 132 included patients that were admitted with provisional diagnosis of acute appendicitis and then their surgery was performed in General Surgery Unit, Hayatabad Medical Complex Peshawar, Pakistan from 1st January 2022 to 31st July 2022. Final diagnosis was confirmed by histopathology report and scores were correlated with final report. Cut off value of score >7, >5 and >7.5 were set for Alvarado, AIR and RIPASA score, respectively according to previous literature. Statistics analysis was done for all 3 scoring systems on SPSS version 23.

**Results:**

Of 132 patients, there were n = 79(59.8%) males and n = 53(40.2%) females. Mean age was 24 years (SD ± 11.6) with youngest patient of 9 years and oldest one was 70 years old. Negative Appendectomy rate was 8.3%(n = 11). RIPASA score was superior to AIR and Alvarado score in Sensitivity, NLR, Accuracy and Area under the Curve. AIR score performed better in specificity, NPV, PLR compared to RIPASA and Alvarado score.

**Conclusion:**

RIPASA score is an overall better scoring system in diagnosing acute appendicitis in compared to Alvarado and AIR score.

## 1 Introduction

With the lifetime risks of 8.6% in males and 6.7% in females, appendicitis is one of the most prevalent causes of acute abdominal pain in adults and children [1]. Diagnostic challenges arise when patients comes with aberrant findings, that results in negative appendectomies. Negative appendectomies are performed at a rate of 8–35%, with higher rates (up to 45%) seen in women of reproductive age [2]. Negative appendectomy although thought to be a benign procedure but it is associated with increase length of hospital stay and cost [3]. Higher morbidity and mortality has been associated with delayed presentation of acute appendicitis. To avoid this issue, surgeons adopt early surgical approach based on their experiences and patients presentation [4]. Despite advancements in technologies, the diagnosis of appendicitis primarily relies on the patient’s medical history and physical examination [5]. Different scoring systems were established in order to assist in the diagnosis of acute appendicitis [6]. Alvarado score, although used widely, has several disadvantages. It does not include C - reactive protein (CRP) as a variable, despite several studies demonstrating the usefulness of CRP in evaluating patients with acute appendicitis [7]. CRP is an essential variable in the Acute Inflammatory Response (AIR) score. The AIR score may decrease unnecessary radiological and surgical interventions [8]. Compared to the Alvarado or Modified Alvarado scores, the Raja Isteri Pengiran Saleh (RIPASA) score, a new diagnostic scoring system, has shown to have significantly higher sensitivity, specificity, and diagnostic accuracy in acute appendicitis compared to other two scoring system. It is especially true when the last two scores were used in an Asian population. It includes factors which are not included in Alvarado score such as age, gender, urinalysis, guarding, Rovsing sign, and Asian origin [9]. The treatment of patients with suspected acute appendicitis remains challenging, and the ideal care plan is still being debated, despite using Computerize Tomography (CT), Ultrasonography (USG), and diagnostic laparoscopy. The histopathologic examination is considered as the gold standard but it is expensive and is not readily available [10].

The rationale of this study is to compare the efficacy of Alvarado, AIR, and RIPASA score in the diagnosis of acute appendicitis. This is the first study that is conducted in our region and results of this study will be used to develop future research and policy recommendations considering acute appendicitis diagnosis.

### 1.1 Objectives

To compare the diagnostic ability of Alvarado Score, AIRS, and RIPASA score, in establishing the diagnosis of acute appendicitis.

### 1.2 Tool of assessment

AIR, Alvarado, and RIPASA scores will be used for the assessment of patients for diagnosis of acute appendicitis (Tables 1–3).

**Table 1:** Alvarado score.

Feature	Score
Migratory Pain	1
Anorexia	1
Nausea	1
Tenderness in right lower quadrant	2
Rebound Tenderness	1
Elevated Temperature	1
Leukocytosis	2
Shift of white blood cells to left	1
**Total**	**10**

Sum of Score <5: Appendicitis unlikely.

Sum of Score 5 or 7: Suspected Appendicitis.

Score >7: Appendicitis Most Likely.

**Table 2:** Appendicitis inflammatory response score.

Variables	Score
Vomiting	1
Pain in Right Inferior fossa	1
Rebound Tenderness Light	1
Or Muscular Defense Medium	2
Strong	3
Body Temperature >38.5	1
Polymorphonuclear leukocytes 70–84%	1
>85%	2
WBC count 10–14.9	1
>15.0	2
CRP concentration 1–4.9 mg/L	1
>5 mg/L	2
**Total**	**12**

1.0–4 score = Low probability Appendicitis.

2.5–7 = Suspected Appendicitis.

3.9–12 = High Probability Appendicitis.

**Table 3:** RIPASA score.

Characteristics		Score
Gender	Female	0.5
	Male	1.0
Age	Age < 39.9	1.0
	Age > 40 years	0.5
Symptoms	RIF pain	0.5
	Pain Migration to RIF	0.5
	Anorexia	1.0
	Nausea and vomiting	1.0
Duration of Symptoms	Duration of Symptoms < 48 h	1.0
	Duration of Symptoms > 48 h	0.5
Signs	RIF tenderness	1.0
	RIF Guarding	2.0
	Rebound Tenderness	1.0
	Rovsing Sign	2.0
	Fever >37 - <39	1.0
Investigations	Leukocytosis	1.0
	Negative Urine Analysis	1.0
Other	Foreign Nationality	1.0
**Total Score**		**17.5**

^a^[10] < 5 points (unlikely, patient observation).

^b^[10] 5–7 points (low probability, emergency room observation, abdominal ultrasound).

^c^[10] 7.5–11.5 points (high probability, surgical evaluation, and preparation for appendectomy) d) > 12 points (appendicitis diagnosis, appendectomy).

## 2 Methods

After approval from institutional review board (Approval No: 904), a hospital-based observational, prospective study was conducted on 132 included patients that were admitted with a provisional diagnosis of acute appendicitis at the General Surgery unit, Hayatabad Medical Complex Peshawar, from 1st January 2022 to 31st July 2022. The sample size of 132 was calculated using WHO calculator keeping in view the negative appendectomy rates of 9.6%^17^(p value of 0.005 and confidence interval of 95%). Based on exclusion criteria n = 32 patients were excluded from study. The sampling technique was nonprobability and convenient. Informed consents were taken from patients after briefing about study. All patient undergone routine laboratory and ultrasound investigation. No CT scan was done for any of the patient. Surgical residents calculated the results of the Alvarado, AIR, and RIPASA scoring system in the Medcalc application, and data were entered in pre-prepared forms on Google forms. The specialist surgeon on duty decided to operate on admitted patients. Histopathology samples were sent for confirmation of acute appendicitis, the results were correlated with preoperative scores. Data collected were transferred to Excel Spreadsheet, and analysis was done by SPSS version 23. Cut off value of score >7, >5 and >7.5 were set for Alvarado, AIR and RIPASA score, respectively, according to previous literature (9). Sensitivity, specificity, positive predictive value (PPV) negative predictive value (NPV), positive likelihood ratio (PLR) and negative likelihood ratio (NLR) and area under the curve (AUC) were calculated for every scoring system and were compared.

### 2.1 Inclusion criteria

All patients presented with pain in the right iliac fossa admitted as a provisionally diagnosed case of acute appendicitis irrespective of their gender, age, and ethnicity.

### 2.2 Exclusion criteria

Patients having nephrolithiasis, patients with lump in right Iliac fossa, patients having any other pathology found intraoperatively, immunocompromised patients, pregnant patients and those patients who didn’t give informed consent were excluded from the study.

## 3 Results

In the study, there were n = 79(59.8%) male and n = 53(40.2%) female. The mean age was 24 years (SD ± 11.6), with the youngest patient 9 years and the oldest one 70 years old. The negative Appendectomy rate was 8.3% (n = 11). Of 132 samples, histopathology showed acutely inflamed appendicitis in 69.7% (n = 92), Gangrenous Appendicitis in 3% (n = 4), and Perforated Appendicitis in 18.9% (n = 25) of samples. The mean values of the score were 8.144(SD ± 1.41), 7.69(SD ± 1.82), and 11.68(SD ± 2.1) for Alvarado, AIR, and RIPASA scores, respectively.

At >7 cut off value sensitivity of 88.4%, specificity of 63.6%, PPV of 96.4%, NPV 33.3%, PLR of 2.43, NLR of 0.18 and accuracy of 86.3% was noted for Alvarado score.

Sensitivity of 77.7%, specificity of 81.8%, PPV of 97.9%, of NPV 25%, PLR of 4.27, NLR 0.27, and accuracy of 78.03% was noted for AIR score at a cutoff value of >5.

Sensitivity 96.7%, specificity 72.7%, PPV of 97.5%, NPV of 66.7%, PLR of 3.54, NLR of 0.05 and accuracy of 94.6% was noted for RIPASA score at cutoff value of >7.5. The summary of findings is tabulated in Table 4.

**Table 4:** Comparison of Different Diagnostic Predictive Values of Alvarado, AIR and RIPASA score.

TESTS	Sensitivity	Specificity	PPV	NPV	PLR	NLR	AUC	Accuracy
Alvarado Score Cut off > 7	88.4%	63.6%	96.4%	33.3	2.43	0.18	.782	86.3
AIR Score Cut off > 5	77.7%	81.8%	97.9%	25	4.27	0.27	.790	78.03
RIPASA Score Cut off > 7.5	96.7%	72.7%	97.5%	66.7	3.54	0.05	0.856	94.6

AIR, acute inflammatory response; RIPASA, Raja Isteri Pengiran Anak Saleha Appendicitis.

PPV, Positive Predictive Value; NPV, Negative Predictive Value.

PLR, Positive Likelihood Ratio; NLR, Negative Likelihood Ratio.

AUC, Area under the curve.

**Fig. 1:**
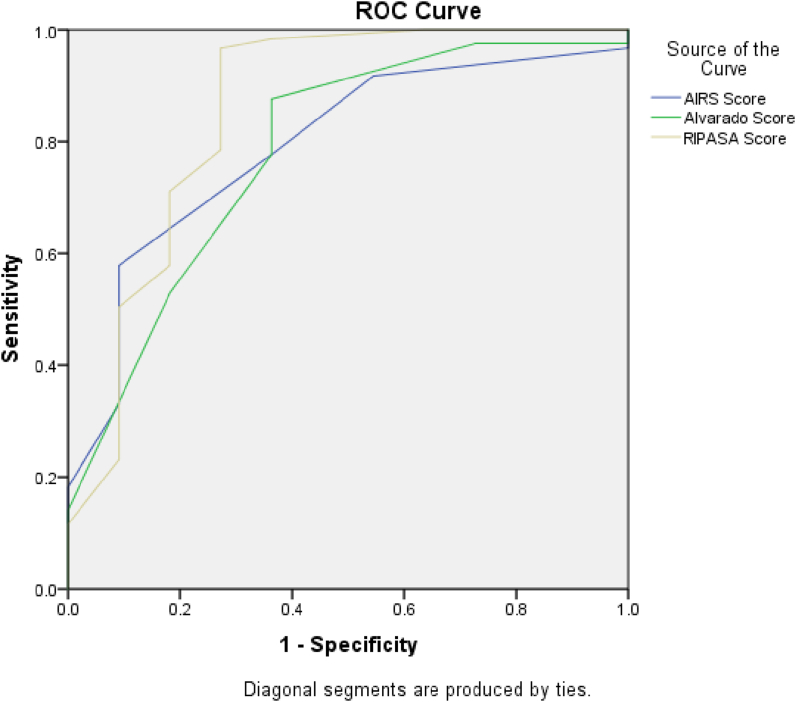
Auc for scoring systems.

When the Receiver operator curve (ROC) was plotted, AUC of 0.782, 0.790, and 0.856 was noted for Alvarado, AIR, and RIPASA scores, respectively (Fig. 1). The difference in AUC of 7.4% between Alvarado and RIPASA and 6.6% of the difference in AUC between RIPASA and AIR score was noted at a p-value of 0.001. The above findings show that Alvarado misdiagnosed 7.4% of people compared to RIPASA score, and 6.6% of people were misdiagnosed by AIR score compared to RIPASA score.

Strong correlation was noted between The RIPASA and Alvarado scores with a Pearson’s coefficient of 0.58. There was weak correlation between AIR and Alvarado scores with a Pearson’s coefficient of 0.57. There was very weak correlation between RIPASA and AIR scores with a Pearson’s coefficient of 0.39.

## 4 Discussion

Acute appendicitis is one of the most common surgical emergencies encountered by surgical residents in their residency period. Diagnosis of acute appendicitis is mainly based on clinical findings, which is why diagnosing it is quite challenging. CT scan abdomen and pelvis with contrast is commonly used to confirm the diagnosis of acute appendicitis due to its high sensitivity (94%) and specificity (95%) compared to other investigations [10]. Until this date, clinical examination is widely used modality in diagnosing appendicitis in many periphery centers where there is non-availability of CT scan. Due to this, there are higher chances of misdiagnosing acute appendicitis, resulting in a negative appendectomies of around 15–30% [11,12]. Ultrasound and CT scan, due to their high cost, can increase the burden of cost on health care setups [13]. The most widely used scoring system, i.e. the Alvarado score, developed in 1986, can help to diagnose acute appendicitis [10]. In the western population, it has high sensitivity and specificity, but in literature, it has low diagnostic power in the Asian population [12,14]. For the Asian population, a new scoring system is introduced, the RIPASA score, which has 14 fixed variables, and those variables have some parameters that are unique to that population [10].

The negative appendectomy rate in our study was 8.3% (n = 11) compared to 12%, 13% 15% and 28.5% reported in other studies respectively [6,[15], [16], [17]].

In our study, the sensitivity of the RIPASA score (94%) was far better than Alvarado (88%) and AIR score (77%). The same findings were reported in another study by Chong et al. and Walia et al. [9,10]. These results are opposite to study conducted by Chishti et al., which reported AIR score has better sensitivity compared to RIPASA and Alvarado score [18].

In our study Specificity of AIR score was better than the RIPASA and Alvarado score, which was comparable to findings of other studies [6,19]. These findings do not correlate with the findings reported in other studies [20,21]. The PPV and NPV of RIPASA were overall better than the AIR and Alvarado Score, which was comparable to the study of Walia et al. [10].

In our study, the RIPASA score has the highest diagnostic accuracy and AUC compared to the AIR and Alvarado scores. Identical findings were noted in other studies [10,18]. In our study Alvarado score and RIPASA score had diagnostic accuracy above 80% but AIRS was below 80%. These results don’t correlate with the results of other study which reported diagnostic accuracy of all three scoring systems above 80% [22].

To summarize, the area under the ROC curve for the RIPASA and AIR scoring systems was larger than it was with the Alvarado system. The RIPASA score is overall a better scoring system in categorizing patients with suspected appendicitis and may reduce the need for diagnostic imaging and negative appendectomy rates.

Our study is subjected to few limitations. First, Sample size can be increased to make case for strong association. Second, acute appendicitis diagnosis was based on clinical evaluation of consultant on duty and it might lead to potential subjective bias. Third, during calculating the scoring system by surgical residents the potential source of subjective bias arises when the resident expertise giving a score to abdominal exploration signs.

Despite limitations, this is the only prospective study conducted in our region and it opens a new chapter in our region to do further studies on this topic in order to find out firm associations and efficacy of scoring systems for diagnosing one of the most common surgical problem.

## 5 Conclusion

RIPASA is an overall better diagnostic scoring system than Alvarado and AIR scores in predicting acute appendicitis. The score also can be helpful to setups where radiological investigations are not readily available.

## Author contribution statement

Muhammad Zeb: Conceived and designed the experiments; Performed the experiments; Analyzed and interpreted the data; Contributed reagents, materials, analysis tools or data; Wrote the paper.

Sabir Khan Khattak: Analyzed and interpreted the data; Contributed reagents, materials, analysis tools or data.

Maryam Samad; Syed Shayan Shah; Syed Qasim Ali Shah; Abdul Haseeb: Performed the experiments; Contributed reagents, materials, analysis tools or data.

## Funding statement

This research did not receive any specific grant from funding agencies in the public, commercial, or not-for-profit sectors.

## Data availability statement

Data will be made available on request.

## Declaration of interest’s statement

The authors declare that they have no known competing financial interests or personal relationships that could have appeared to influence the work reported in this paper.

## Additional information

No additional information is available for this paper.

## References

[1] Snyder M.J., Guthrie M., Cagle S. (2018). Acute appendicitis: efficient diagnosis and management. Am. Fam. Physician.

[2] Rao P.M., Rhea I.I., Novelline R.A. (1998). Effect of computed tomography of the appendix on treatment of patients and use of hospital resources. NEJM.

[3] Mock K., Lu Y., Friedlander S., Kim D.Y., Lee S.L. (2016). Misdiagnosing adult appendicitis: clinical, cost, and socioeconomic implications of negative appendectomy. Am. J. Surg..

[4] Pitman-Waller V.A., Myers J.G., Stewart R.M., Dent Dl (2000). Appendicitis: why so complicated? Analysis of 5755 consecutive appendectomies. Am. Surg..

[5] Flum D.R., Morris A., Koepsell T., Dellinger E.P. (2001). Has misdiagnosis of appendicitis decreased over time? A population-based analysis. JAMA.

[6] Karami M.Y., Niakan H., Zadebagheri N., Mardani P., Shayan Z., Deilami I. (2017). Which one is better? Comparison of the acute inflammatory response, Raja Isteri Pengiran Anak Saleha appendicitis and Alvarado scoring systems. Ann Coloproctol.

[7] Andersson R.E. (2004). Meta-analysis of the clinical and laboratory diagnosis of appendicitis. Br. J. Surg..

[8] Yeşiltaş M., Karakaş D.Ö., Gökçek B., Hot S., Eğin S. (2018). Can Alvarado and Appendicitis Inflammatory Response scores evaluate the severity of acute appendicitis?. Ulus Travma Acil Cerrahi Derg.

[9] Chong C.F., Adi M.I., Thien A., Suyoi A., Mackie A.J., Tin A.S. (2010). Development of the RIPASA score: a new appendicitis scoring system for the diagnosis of acute appendicitis. Singap. Med. J..

[10] Walia D.S.W., Shankar N., Singla A., Najmi H., Kaur M. (2022). A comparative study of Alvarado, Ripasa and Airs scoring systems in the diagnosis of acute appendicitis. Eur. J. Mol. Clin. Med..

[11] Chan I., Bicknell S.G., Graham M. (2005). Utility and diagnostic accuracy of sonography in detecting appendicitis in a community hospital. AJR Am. J. Roentgenol..

[12] Flum D.R., McClure T.D., Morris A., Koepsell T. (2005). Misdiagnosis of appendicitis and the use of diagnostic imaging. J. Am. Coll. Surg..

[13] Livingston E.H., Woodward W.A., Sarosi G.A., Haley R.W. (2007). Disconnect between incidence of nonperforated and perforated appendicitis: implications for pathophysiology and management. Ann. Surg..

[14] Terasawa T., Blackmore C.C., Bent S. (2004). Systematic review: computed tomography and ultrasonography to detect acute appendicitis in adults and adolescents. Ann. Intern. Med..

[15] Hale D.A., Molloy M., Pearl R.H., Schutt D.C., Jaques D.P. (1997). Appendectomy: a contemporary appraisal. Ann. Surg..

[16] Andersson R., Hugander A., Thulin A. (1992). Diagnostic accuracy and perforation rate in appendicitis: association with age and sex of the patient and with appendicectomy rate. Eur. J. Surg..

[17] Memon Z.A., Irfan S., Fatima K., Iqbal M.S., Sami W. (2013). Acute appendicitis: diagnostic accuracy of Alvarado scoring system. Asian J. Surg..

[18] Chisthi M.M., Surendran A., Narayanan J.T. (2020). RIPASA and air scoring systems are superior to Alvarado scoring in acute appendicitis: diagnostic accuracy study. Ann. Med. Surg..

[19] Bolívar-Rodríguez M.A., Osuna-Wong B.A., Calderón-Alvarado A.B., Matus-Rojas J., Dehesa-López E., de Jesús Peraza-Garay F. (2018). Comparative analysis of diagnostic scales of acute appendicitis: Alvarado, RIPASA and AIR. Cirugía Cir..

[20] Chisthi M.M., Surendran A., Narayanan J.T. (2020). RIPASA and air scoring systems are superior to Alvarado scoring in acute appendicitis: diagnostic accuracy study. Ann. Med. Surg. (Lond.).

[21] Scott A.J., Mason S.E., Arunakirinathan M. (2015). Risk stratification by the appendicitis inflammatory response score to guide decision-making in patients with suspected appendicitis. Br. J. Surg*.*.

[22] Alhamdani Y.F., Rizk H.A., Algethami M.R., Algarawi A.M., Albadawi R.H., Faqih S.N. (2018). Negative appendectomy rate and risk factors that influence improper diagnosis at King Abdulaziz University Hospital. Mater. Soc. Med..

